# Effects of Short-Term Nighttime Carbohydrate Restriction Method on Exercise Performance and Fat Metabolism

**DOI:** 10.3390/nu16132138

**Published:** 2024-07-04

**Authors:** Takumi Sakamoto, Shin-ya Ueda, Hidehiro Nakahara

**Affiliations:** 1 Graduate School of Health Science, Morinomiya University of Medical Sciences, Osaka 559-8611, Japan; st.19961228@gmail.com; 2 Faculty of Education, Gifu University, Gifu 501-1193, Japan; ueda.shin-ya.n3@f.gifu-u.ac.jp; 3 Department of Acupuncture, Morinomiya University of Medical Sciences, Osaka 559-8611, Japan

**Keywords:** carbohydrate metabolism, aerobic exercise, body composition, dietary restrictions

## Abstract

Background: The sleep-low method has been proposed as a way to sleep in a low-glycogen state, increase the duration of low glycogen availability and sleep and temporarily restrict carbohydrates to improve exercise performance. However, long-term dietary restriction may induce mental stress in athletes. Therefore, if it can be shown that the effects of the sleep-low method can be achieved by restricting the carbohydrate intake at night (the nighttime carbohydrate restriction method), innovative methods could be developed to reduce weight in individuals with obesity and enhance athletes’ performance with reduced stress and in a shorter duration when compared with those of previous studies. With this background, we conducted a study with the purpose of examining the intervention effects of a short-term intensive nighttime carbohydrate restriction method. Methods: A total of 22 participants were recruited among university students participating in sports club activities. The participants were assigned at random to groups, including a nighttime carbohydrate restriction group of 11 participants (6 males, 5 females; age 22.3 ± 1.23) who started a carbohydrate-restricted diet and a group of 11 participants (5 males, 6 females; age 21.9 ± 7.9) who continued with their usual diet. The present study had a two-group parallel design. In the first week, no dietary restrictions were imposed on either group, and the participants consumed their own habitual diets. In the second week, the total amount of calories and carbohydrate intake measured in the first week were divided by seven days, and the average values were calculated. These were used as the daily calorie and carbohydrate intakes in the second week. Only the nighttime carbohydrate restriction group was prohibited from consuming carbohydrates after 4:00 p.m. During the two-week study period, all participants ran for one hour each day before breakfast at a heart rate of 65% of their maximum heart rate. Results: The results obtained from young adults participating in sports showed significant differences in peak oxygen consumption ($\overset{\cdot }{V}$O2peak), work rate max, respiratory quotient (RQ), body weight and lean body mass after the intervention when compared with before the intervention in the nighttime carbohydrate restriction group (*p* < 0.05). Conclusions: Our findings suggest that the nighttime carbohydrate restriction method markedly improves fat metabolism even when performed for a short period. This method can be used to reduce body weight in individuals with obesity and enhance athletes’ performance. However, it is important to consider the intake of nutrition other than carbohydrates.

## 1. Introduction

The advancement of knowledge on the relationship between nutrition and exercise in recent years has raised expectations for novel possibilities to improve the performance of athletes. Previous studies have indeed indicated that nutritional intake has the potential to influence optimal training and competition performance positively [1,2]. The key to nutritional strategies for endurance athletes is to integrate two apparently paradoxical approaches. The first approach is to optimize performance by optimizing energy utilization, which is achieved by promoting the glucose metabolism response (carbohydrate availability) before and during prolonged high-intensity exercise [3]. The second approach is to improve adaptation to training by optimizing the intracellular environment of the skeletal muscle through carbohydrate restriction, the so-called train-low approach [4,5,6,7,8,9].

Carbohydrate utilization is an important factor in the performance of prolonged exercise and intermittent high-intensity exercise [10]. However, it should be noted that high-intensity exercise and carbohydrate consumption due to long exercise occur beyond the stored amount because of the amount of glycogen stored in the body. In endurance sports, the reduction or depletion of glycogen in the body is a cause of poor performance [11,12]. For this reason, the ingestion of carbohydrates before exercise, during exercise and in the recovery period after exercise is recommended [13]. The prevention of carbohydrate depletion by these strategies is related to the attenuation of fatigue and the maintenance of endurance [14,15,16], and some studies have shown that an increase in carbohydrate supplementation improves performance [17,18,19]. Therefore, it is considered important to train in a state in which glycogen is always replenished during exercise. However, more recent studies have reported that periodically reducing carbohydrate availability further enhances adaptations to endurance training [20]. Several studies have reported the upregulated expression of markers of training adaptation (an increase in fat oxidation and the activation of oxidative enzymes) when training in a state of low muscle glycogen compared with training in a state of normal muscle glycogen stores [4,5,6,7,8,9]. However, despite the observed changes in molecular and cellular markers, there is no clear evidence that these changes translate to an improved performance. Therefore, the sleep-low method has been proposed as a way to sleep in a low-glycogen state, increase the duration of low glycogen availability and sleep and temporarily restrict carbohydrates to improve exercise performance [21,22,23]. The objective of the sleep-low method is to increase mitochondria in the body. Since the energy required for exercise is mainly generated by mitochondria, increasing the number of mitochondria is indispensable for improving exercise performance. To increase the number of mitochondria, it is necessary to activate adenosine monophosphate kinase (AMPK), an enzyme that shows enhanced production in endurance exercise. However, studies have found that AMPK activity is inhibited by glycogen [24] and enhanced more by exercising in a low-glycogen state than in the usual glycogen state [25]. Therefore, the sleep-low strategy aims to activate AMPK by restricting carbohydrates from the evening meal, going to bed in a state of low glycogen stores in the body and then performing aerobic exercise the next morning before breakfast. However, some reports have shown that high-intensity interval training (HIIT) in a low-glycogen state and a low-carbohydrate state reduces training intensity [5,9]. Thus, when attempting to improve performance by changing the method of carbohydrate intake, it is important to ensure that the training objectives are not compromised.

Carbohydrate-restricted diets are used not only by persons with obesity for weight reduction but also by general people as an easy method to lose weight. Furthermore, other than persons with obesity and general people, research has been conducted on athletes regarding the effect of the dietary periodization strategy on weight loss and performance improvement. A study involving 21 triathletes reported that the sleep-low strategy may contribute to an improvement in exercise performance, as evidenced by an improvement in 10 km running performance and a decrease in the rated perceived exertion (RPE) score during constant exercise in those participants [22]. This report also indicated that the strategy was effective in reducing body weight and body fat percentage by increasing the fat utilization rate. These results are not only beneficial for improving the performance of athletes but they could also be beneficial for exercise therapy aiming at weight loss. However, in the previous study, dietary restriction was implemented four times a week for only three weeks out of concern that long-term dietary restriction may induce mental stress in athletes. Indeed, previous studies have shown that exercise performance is higher in participants with lower stress levels and that the risk of injury is approximately twofold higher during periods of increased stress when compared with the usual stress level, indicating a close association between stress and exercise [26,27]. Hence, even if the sleep-low strategy improves the functions of the body, the stress incurred may cause a decrease in performance. If it can be shown that the effects of the sleep-low method can be achieved by restricting the carbohydrate intake at night (the nighttime carbohydrate restriction method), innovative methods could be developed to reduce weight in individuals with obesity and enhance athletes’ performance via reduced stress and a shorter duration when compared with the methods in previous studies. With this background, we conducted a study with the purpose of examining the intervention effects of a short-term intensive nighttime carbohydrate restriction method.

## 2. Materials and Methods

### 2.1. Ethical Approval

The present study was approved by the Human Subjects Committee of Morinomiya University of Medical Sciences (No. 2022-085). All procedures in the present study conformed to the ethical principles of the Declaration of Helsinki. This study was not registered in a database. All the procedures, potential risks and purposes of this study were explained thoroughly to each subject before the initiation of experiments. Informed consent was obtained from each subject prior to participation in the experiment.

### 2.2. Participants

A total of 22 participants were recruited among university students participating in sports club activities. The participants were assigned at random to groups, including a nighttime carbohydrate restriction group of 11 participants (6 males, 5 females; age 22.3 ± 1.23, height 166.8 ± 6.2 cm) who began a carbohydrate-restricted diet and a group of 11 participants (5 males, 6 females; age 21.9 ± 7.9, height 165.8 ± 7.9 cm) who continued their usual diet. The present study had a two-group parallel design. The random assignment procedure was performed by a third party not involved in this study.

### 2.3. Experimental Protocol

**Figure 1:**
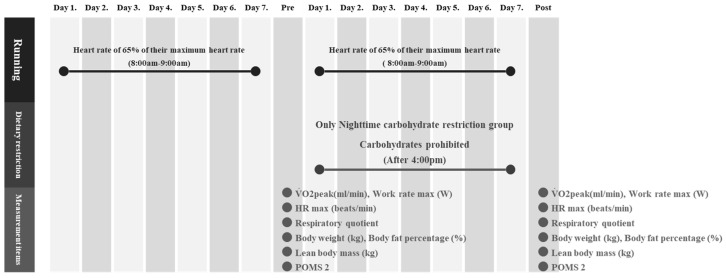
Running, dietary restriction and measurement item protocols for the nighttime carbohydrate restriction and control groups during the 2-week experimental period.

The experimental protocol is shown in Figure 1.

(1) Dietary restriction (Calorie intake, Carbohydrate intake)

In the first week, no dietary restrictions were imposed on either group, and the participants consumed their habitual diet. Calorie and carbohydrate intakes were measured and recorded using an AI health app (Calomama, Link, and Communication). Daily calorie and carbohydrate intakes in the second week were calculated by dividing the total intakes from the first week by seven. Although only the nighttime carbohydrate restriction group was prohibited from consuming carbohydrates after 4:00 p.m., the daily calorie and carbohydrate intakes were the same in both groups. All participants were also asked to refrain from consuming alcohol or caffeine during the experimental period.

(2) Running

In a previous study, Marquet et al. [22] examined the impact of a chronic dietary periodization strategy on endurance performance in athletes. In their study, low-intensity cycling exercise was performed, which consisted of 60 min of cycling at an intensity corresponding to 65% of maximal aerobic power. Accordingly, the exercise intensity in the present study was set to 60 min at 65% of the maximal heart rate (154.3 ± 2.8 beats/min). The exercise intensity was determined by the Karvonen method (Karvonen method: target heart rate = [(220 − age) − resting heart rate] · 0.65 + resting heart rate]). Heart rate monitors were attached to the wrist during the running period in all participants to monitor the heart rate response. Outdoor running was performed for 1 h between 8:00 a.m. and 9:00 a.m.

### 2.4. Measurement Items and Methods

Pre- and post-measurements were performed 24 h after the end of the running period to eliminate the effects of running and/or carbohydrate restrictions.

(1) Peak oxygen consumption ($\overset{\cdot }{V}$O2peak), maximum watts (Work rate max) and maximum heart rate (HRmax)

$\overset{\cdot }{V}$O2peak was measured using a bicycle ergometer (Aerobike 75XL, Combi Co., Ltd., Tokyo, Japan). After 3 min of rest while sitting, the participants warmed up at a load of 20 watts for 6 min. Thereafter, the participants performed a ramp load test of 20 watts/min and were instructed to maintain a pace of 60 revolutions per minute (rpm). Exercise was terminated when the pace of 50 rpm could not be maintained. Ventilation volume was measured by the breath-by-breath method using an expired gas analyzer (AE-310, Minato Medical Science Co., Ltd., Osaka, Japan). The data were averaged over 5 s and the peak value was calculated as $\overset{\cdot }{V}$O2peak. Heart rate was measured using a heart rate monitor (POLAR V800, Polar Electro, Kempele, Finland).

(2) Respiratory quotient

The respiratory quotient (RQ) was measured using an expired gas analyzer. After 3 min of rest while sitting, the participants exercised at a load of 20 watts for 6 min, and the RQ was measured for the 2 min period before the end.

(3) Body composition

Body weight and body fat percentage were measured by the impedance method using a body composition monitor, HBF-701 (Omron Healthcare Co., Ltd., Kyoto, Japan). Lean body mass was calculated from the body composition measurements.

(4) Mood profile

The mood profile was measured using the Profile of Mood States (POMS) scale (POMS 2 for Adults, Full Scale, Kaneko Shobo; in Japanese). The eight test items were Anger—Hostility, Confusion—Bewilderment, Depression—Dejection, Fatigue—Inertia, Tension—Anxiety, Vigor—Activity, Friendliness and the Total Mood Disturbance Score.

### 2.5. Statistical Analysis

All data are presented as the mean ± standard deviation. The POMS 2 score, $\overset{\cdot }{V}$O2peak, HRmax, work rate max, RQ, body weight, body fat percentage and lean body mass were analyzed by two-way analysis of variance (ANOVA) (group × time). When an interaction was observed, a simple main effects test was conducted. When interaction was not observed, the presence or absence of a main effect was tested. Bonferroni’s method was used as the post hoc test. In this study, a *p*-value less than 0.05 was considered to indicate a statistically significant difference.

## 3. Results

### 3.1. Calorie Intake

A comparison of calorie intakes before and after the intervention in the nighttime carbohydrate restriction and control groups is shown in Table 1. Two-way ANOVA detected no interaction effect between the group and time, and there was no significant difference in calorie intake after the intervention versus before or between groups.

### 3.2. Carbohydrate Intake

A comparison of the carbohydrate intake before and after the intervention in the nighttime carbohydrate restriction and control groups is shown in Table 1. Two-way ANOVA detected no interaction effect between group and time, and there was no significant difference in carbohydrate intake after the intervention versus before or between groups.

### 3.3. POMS 2 Score

The POMS 2 scores before and after the intervention in the nighttime carbohydrate restriction and control groups are shown in Table 2. Two-way ANOVA detected no interaction between group and time, and there were no significant differences in the scores of the POMS 2 items after the intervention versus before or between groups.

### 3.4. $\overset{\cdot }{V}$O2peak

**Figure 2:**
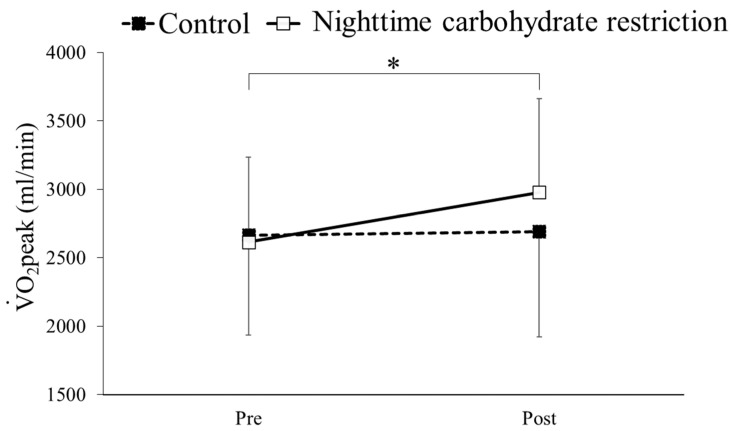
Comparison of $\overset{\cdot }{V}$O2peak in a maximal exercise test before (Pre) and after (Post) intervention in a control group and nighttime carbohydrate restriction group. * Significant difference.

The $\overset{\cdot }{V}$O2peak results before and after the intervention in the nighttime carbohydrate restriction and control groups are shown in Figure 2. Two-way ANOVA of the $\overset{\cdot }{V}$O2peak results detected a significant interaction between the group and time (F = 10.01, *p* < 0.05). A simple main effects test was conducted, and a significant difference in the $\overset{\cdot }{V}$O2peak results before and after the intervention was observed in the nighttime carbohydrate restriction group. Multiple comparison analysis showed significantly higher $\overset{\cdot }{V}$O2peak results after the intervention compared to before the intervention in the nighttime carbohydrate restriction group (*p* < 0.05).

### 3.5. HRmax

**Figure 3:**
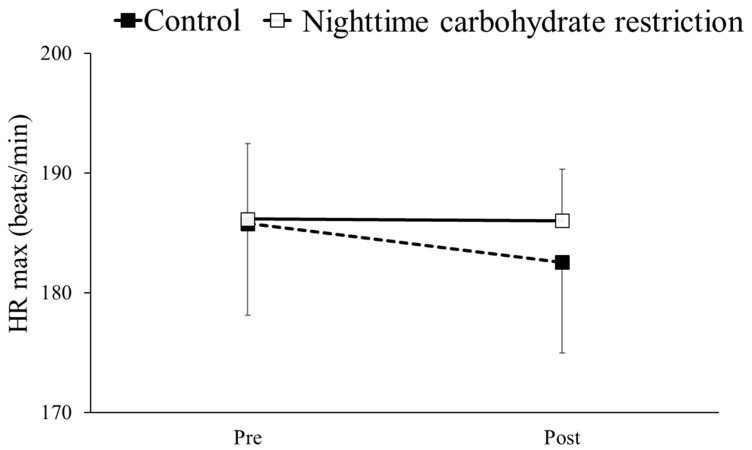
Comparison of maximum heart rate in a maximal exercise test before (Pre) and after (Post) intervention in a control group and nighttime carbohydrate restriction group.

The HRmax results before and after the intervention in the nighttime carbohydrate restriction and control groups are shown in Figure 3. Two-way ANOVA detected no interaction between the group and time, and there were no significant differences in HRmax results after the intervention versus before or between groups.

### 3.6. Work Rate Max

**Figure 4:**
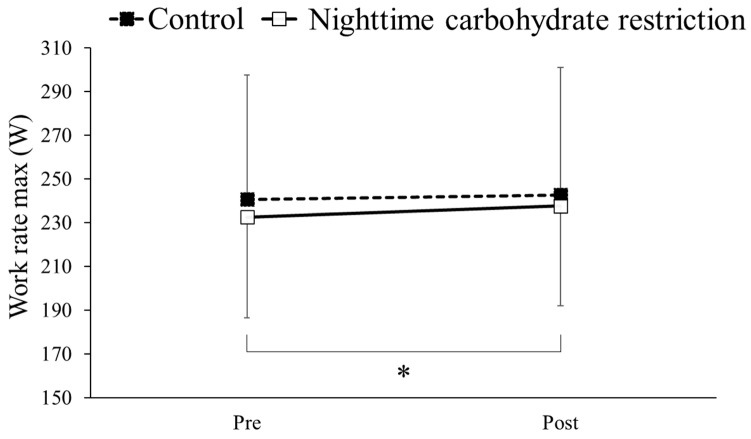
Comparison of work rate max in a maximal exercise test before (Pre) and after (Post) intervention in a control group and nighttime carbohydrate restriction group. * Significant difference.

The work rate max results before and after the intervention in the nighttime carbohydrate restriction and control groups are shown in Figure 4. Two-way ANOVA detected no significant interaction effect between the group and time. When the main effect of time was tested, a significant difference was observed. Multiple comparison analysis showed a significantly higher work rate max after the intervention compared to before the intervention in the nighttime carbohydrate restriction group (*p* < 0.05).

### 3.7. RQ

**Figure 5:**
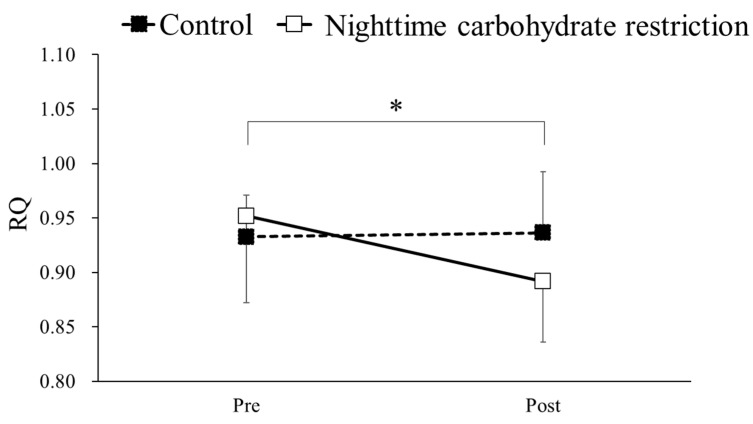
Comparison of respiratory quotient (RQ) in a maximal exercise test before (Pre) and after (Post) intervention in a control group and nighttime carbohydrate restriction group. * Significant difference.

The RQs before and after the intervention in the nighttime carbohydrate restriction and control groups are shown in Figure 5. Two-way ANOVA detected a significant interaction between the group and time (F = 7.04, *p* < 0.05). A simple main effects test was conducted, and a significant difference in RQs before and after the intervention was observed in the nighttime carbohydrate restriction group. Multiple comparison analysis showed a significantly lower RQ after the intervention compared to before the intervention (*p* < 0.05).

### 3.8. Body Weight

A comparison of body weights before and after the intervention in the nighttime carbohydrate restriction and control groups is shown in Table 3. Two-way ANOVA detected a significant interaction effect between the group and time (F = 12.64, *p* < 0.05). A simple main effects test revealed a significant difference in body weights before and after the intervention in the nighttime carbohydrate restriction group, and a multiple comparison test showed a significantly lower body weight after the intervention compared to before the intervention in the nighttime carbohydrate restriction group (*p* < 0.05).

### 3.9. Body Fat Percentage

A comparison of body fat percentages before and after the intervention in the nighttime carbohydrate restriction and control groups is shown in Table 3. Two-way ANOVA detected no interaction effect between the group and time, and no significant difference in body fat percentages was found between after the intervention versus before or between groups.

### 3.10. Lean Body Mass

A comparison of lean body masses before and after the intervention in the nighttime carbohydrate restriction and control groups is shown in Table 3. Two-way ANOVA detected a significant interaction effect between the group and time (F = 4.86, *p* < 0.05). A simple main effects test revealed a significant difference in lean body masses before and after the intervention in the nighttime carbohydrate restriction group, and a multiple comparison test showed a significantly lower lean body mass after the intervention compared to before the intervention in the nighttime carbohydrate restriction group (*p* < 0.05).

## 4. Discussion

The purpose of this study was to clarify the intervention effect of a short-term intensive nighttime carbohydrate restriction method. The results obtained from young adults participating in sports showed significant differences in $\overset{\cdot }{V}$O2peak, work rate max, RQ, body weight and lean body mass after the intervention when compared with before the intervention in the nighttime carbohydrate restriction group.

The protocol of this study manipulated the timing of carbohydrate intake and aerobic exercise to specifically alter the energy contribution rate. The novelty of this study lies in successfully verifying the effectiveness of a short-term (one-week) nighttime diet and exercise strategy in comparison to previous studies that focused on carbohydrate restriction in which athletes implemented dietary restrictions four times a week for three weeks [3,28,29,30].

The $\overset{\cdot }{V}$O2peak and work rate max increased in the nighttime carbohydrate restriction group, while such increases were not observed in the control group, showing that the dietary periodization strategy adopted in this study improved endurance. Studies have reported that long-term carbohydrate restriction promotes transcriptional activities of specific genes involved in mitochondria and energy utilization, further contributing to the increase in performance [31,32]. Increased catecholamine activity has also been reported to enhance metabolic adaptation via mechanisms such as increased expression of peroxisome proliferator-activated receptor-gamma coactivator (PGC)-1α, a transcriptional activator [33]. Furthermore, in this study, the RQ decreased in the nighttime carbohydrate restriction group, while not in the control group. A decrease in the RQ during exercise at the same intensity has been reported to indicate an increase in fat oxidation [34]. Presumably, this is due to the effect of the free fatty acid concentration in the blood. A previous study has shown that exercise under low muscle glycogen storage increases the blood free fatty acid concentration [35]. Therefore, we speculate that in the nighttime carbohydrate restriction group of our study, endurance training in a low glycogen state resulted in an increase in mitochondria accompanied by increases in free fatty acid levels in the blood; consequently, the supply and demand for fat utilization in the body increased, leading to the low RQ observed in the nighttime carbohydrate restriction group. Low-intensity exercise under a glycogen-depleted state augments the activation of key cell signaling kinases (AMPK, p38MAPK), transcription factors (p53, PPARδ) and a transcriptional activator (PGC-1α), inducing training adaptations. These adaptations lead to an augmentation of fat metabolism, resulting in higher fat oxidation and an increased muscle glycogen content at rest [4,9,29,36], which is speculated to improve the performance of prolonged exercise.

On the other hand, Riis et al. reported that the sleep-low method over 4 weeks did not translate into any superior sustained adaptations regarding the fat oxidation rate during exercise, endurance performance or intramuscular lipid metabolism in endurance-trained males when compared to a group with high carbohydrate availability [37]. Gejil et al. also indicated that 4 weeks of regular endurance training with periodic restriction of the carbohydrate intake had no superior effects on performance and muscle adaptations in elite endurance athletes [38]. In the present study, the nighttime carbohydrate restriction method in the short-term period of one week induced significant differences in $\overset{\cdot }{V}$O2peak, work rate max, RQ, body weight and lean body mass after the intervention when compared with before the intervention. The inconsistency in the intervention effect of the nighttime carbohydrate restriction method between the present study and these previous studies may be attributed to differences in the interventional period, but further research is needed.

The sleep-low method is now one of the most widely debated topics among athletes, coaches and sports scientists [39]. Previous studies have stated that the low carbohydrate intake during the sleep-low method requires having a lower energy intake [4,6,29,40,41,42]. Areta et al. also indicated that a ‘sleep-low’ model with high-fat meals does not enhance the response of skeletal muscle markers of early adaptation to training, and that it impairs glycemic control the morning after these meals compared to training with low energy availability [43]. The present study demonstrated that participants in the nighttime carbohydrate restriction group had reduced weights, whereas weight loss was not found in the control group. Furthermore, although the body fat percentage was not significantly different between the two groups, a decrease in lean body mass was observed in the nighttime carbohydrate restriction group. Sawaka et al. [44] reported that the lean body mass is composed of about 73% water, while the fat body mass is composed of about 10% water. As such, the bioimpedance values used in the current study are influenced by the hydration status, which may impact the measurement of lean body mass. Therefore, it is possible that a reduced intake of water due to changes in the timing of carbohydrate intake led to a decrease in lean body mass. In addition, carbohydrates and fats are the major nutrients that supply energy during exercise; carbohydrate utilization is high in the early stages of exercise, while fat utilization increases with time during exercise. In the nighttime carbohydrate restriction group of this study, since carbohydrates were restricted from the evening meal of the previous day, carbohydrates were not readily available as the energy source during exercise the next morning. The carbohydrates as an energy source were presumably supplemented by gluconeogenesis, a process of synthesizing glucose from non-carbohydrate substrates [45,46]. In gluconeogenesis, proteins in muscles are transported to the liver, where they are converted to glycogen, and then returned to the muscles; this process requires the breakdown of proteins. Therefore, muscle breakdown due to gluconeogenesis may underlie the decrease in lean body mass observed in this study.

The nighttime carbohydrate restriction method used as the intervention in this study only focused on manipulating the timing of carbohydrate intake during the study. However, the result of a decreased lean body mass after the intervention indicates the need to consider the timing and quantity of other forms of nutritional intake in order to prevent muscle breakdown. Protein consumption after exercise has been reported to increase skeletal muscle protein synthesis and support muscle repair and remodeling [47,48]. In addition, several previous studies have reported that exercise combined with leucine ingestion suppresses muscle atrophy and has a muscle hypertrophy effect [49]. Furthermore, when the amino acid concentrations in the body are low, leucine supplementation does not promote skeletal muscle synthesis, but it can suppress muscle protein degradation, and leucine supplementation has been reported to alleviate muscle atrophy [50,51]. Based on the above findings, as a measure to prevent muscle breakdown, increasing the concentrations of amino acids in the body by consuming proteins before and after exercise is important. In combination with the above measures, it may be necessary to consider the intake of nutrients other than carbohydrates, such as consuming foods rich in leucine. However, in a previous study in mice, feeding the mice with a low-carbohydrate diet for only 2 weeks resulted in a decrease in protein expression of sodium-dependent glucose transporter-1 (SGLT1), which plays a role in the uptake of glucose from the lumen of the small intestine, and these mice showed a marked reduction in insulin secretion after an oral glucose load [52]. Since insulin promotes glucose uptake into the skeletal muscle, it should be noted that carbohydrate restriction may reduce insulin secretion, which may have a negative effect on the recovery of muscle glycogen after exercise.

This study has some limitations. First, we cannot exclude the possibility that the protocol of this study may have induced a placebo effect in the participants. However, for the performance measures, $\overset{\cdot }{V}$O2max and work rate max, the influence of a placebo effect on the body composition could only have been very small. Second, the menstrual cycle of female participants was not considered. Women may gain 1 to 3 kg of weight due to menstruation, caused by the effects of female hormones and premenstrual syndrome. In this study, we cannot rule out the possibility that a trend of remarkable increases in the body weight and body fat percentage after the intervention compared to before, as were observed in some female participants, was due to menstruation. In the future, when including female participants in a study, it is necessary to plan the experimental period considering the menstrual cycle, where the hormonal effects of the menstrual cycle should be taken into account to independently clarify the effects of the nighttime carbohydrate restriction method. Third, the participants’ sports and events were not taken into consideration, and yet we found that, in the nighttime carbohydrate restriction group, there was a difference of more than 2.5 kg in lean body mass between participants with small fluctuations and those with large fluctuations after the intervention compared to before. Therefore, in future studies, we advise considering the characteristics of various sports. Lastly, the persistence of the improved performance produced by the nighttime carbohydrate restriction method is unknown. In this study, we have clarified that the beneficial effects of the intensive nighttime carbohydrate restriction protocol manifest after one week of intervention, but we do not know how long the effects persist. Indeed, previous studies enacted the sleep-low strategy four times a week for three weeks, and the persistence of training effects was not investigated beyond then; hence, this aspect remains unknown. In the future, researchers should further extend the intervention period of the nighttime carbohydrate restriction method and explore the persistence of the effects after the intervention.

## 5. Conclusions

The effects of a short-term intensive nighttime carbohydrate restriction method have been shown to increase lipid metabolism. Increased fat metabolism leads to weight loss and improved endurance. The nighttime carbohydrate restriction method can be used to reduce body weight in individuals with obesity and to improve performance in athletes. However, attention should be paid to the need for nutritional intake support when applying the nighttime carbohydrate restriction method, especially for athletes. In the future, the nighttime carbohydrate restriction method may offer an effective strategy for individuals with different goals.

## Figures and Tables

**Table 1:** Comparison of calorie intake and carbohydrate intake before (Pre) and after (Post) intervention in control group and nighttime carbohydrate restriction group.

	Control Group		Nighttime Carbohydrate Restriction Group	
	(n = 11)		(n = 11)	
	Pre	Post	Pre	Post
Calorie intake (kcal)	2349.5 ± 549.7	2437.8 ± 669.8	2238.5 ± 418.6	2234.5 ± 431.0
Carbohydrate intake (g)	290.2 ± 64.2	287.3 ± 59.6	270.4 ± 69.9	267.3 ± 68.3

Values are presented as mean ± SD.

**Table 2:** Comparison of POMS 2 scores before (Pre) and after (Post) intervention in control group and nighttime carbohydrate restriction group.

	Control Group		Nighttime Carbohydrate Restriction Group	
	(n = 11)		(n = 11)	
	Pre	Post	Pre	Post
Anger—Hostility	39.9 ± 2.6	38.3 ± 0.5	40.3 ± 2.6	42.7 ± 5.4
Confusion—Bewilderment	42.9 ± 6.2	43.1 ± 6.9	47.7 ± 9.4	49.4 ± 10.2
Depression—Dejection	42.4 ± 4.3	42.4 ± 2.4	45.9 ± 4.8	45.4 ± 6.4
Fatigue—Inertia	49.6 ± 8.1	47.8 ± 7.5	53.9 ± 9.5	51.1 ± 9.3
Tension—Anxiety	45.0 ± 8.4	41.0 ± 5.6	46.1 ± 8.9	46.1 ± 7.9
Vigor—Activity	55.3 ± 11.2	53.6 ± 8.7	57.7 ± 11.3	55.1 ± 12.9
Friendliness	56.5 ± 11.4	55.5 ± 9.0	56.4 ± 10.5	54 ± 11.7
Total Mood Disturbance Score	42.3 ± 6.3	41.1 ± 4.5	44.6 ± 7.7	45.3 ± 8.2

Values are presented as mean ± SD.

**Table 3:** Comparison of body composition before (Pre) and after (Post) intervention in a control group and nighttime carbohydrate restriction group.

	Control Group		Nighttime Carbohydrate Restriction Group	
	(n = 11)		(n = 11)	
	Pre	Post	Pre	Post
Body weight (kg)	61.7 ± 11.0	61.5 ± 10.9	62.9 ± 7.7	61.5 ± 7.4 *
Body fat percentage (%)	19.3 ± 4.8	19.0 ± 4.5	19.0 ± 5.2	18.7 ± 6.2
Lean body mass (kg)	50.0 ± 10.3	50.0 ± 10.2	50.9 ± 6.9	50.0 ± 6.6 *

Values are presented as mean ± SD. * Significantly different (*p* < 0.05) from the pre value.

## Data Availability

Informed consent was obtained from all subjects involved in this study.
